# Epigenetics: a new mechanism of regulation of heart failure?

**DOI:** 10.1007/s00395-013-0361-1

**Published:** 2013-06-06

**Authors:** Roberto Papait, Carolina Greco, Paolo Kunderfranco, Michael V. G. Latronico, Gianluigi Condorelli

**Affiliations:** 1 Humanitas Clinical and Research Center, Rozzano (MI), 20098 Italy; 2 Institute of Genetics and Biomedical Research, National Research Council of Italy (CNR), Rozzano (MI), 20098 Italy; 3 University of Milan, Milan, 20100 Italy

**Keywords:** Chromatin remodeling, DNA methylation, Heart failure, Histone modifications, microRNA

## Abstract

Heart failure is a syndrome resulting from a complex genetic predisposition and multiple environmental factors, and is a leading cause of morbidity and mortality. It is frequently accompanied by changes in heart mass, size, and shape, a process known as pathological cardiac remodeling. At the molecular level, these changes are preceded and accompanied by a specific gene expression program characterized by expression of certain ‘fetal’ genes. This re-expression of fetal genes in the adult heart contributes to the development of the syndrome. Therefore, counteracting the gene expression changes occurring in heart failure could be a therapeutic approach for this pathology. One mechanism of gene expression regulation that has gained importance is epigenetics. This review gives an overview of the roles of some epigenetic mechanisms, such as DNA methylation, histone modifications, ATP-dependent chromatin remodeling, and microRNA-dependent mechanisms, in heart failure.

## Introduction

In eukaryotic cells, gene expression is the result of a complex molecular network where several players—regulatory sequences (e.g., promoters and enhancers), RNA polymerases, transcription factors, and co-regulator proteins—interact with one another in controlling transcription and translation of genes. One important regulator of these interactions is epigenetics, a set of mechanisms that influences gene expression without altering the DNA sequence, but that can be transmitted between cell generations nonetheless [5, 24, 44].

**Fig. 1:**
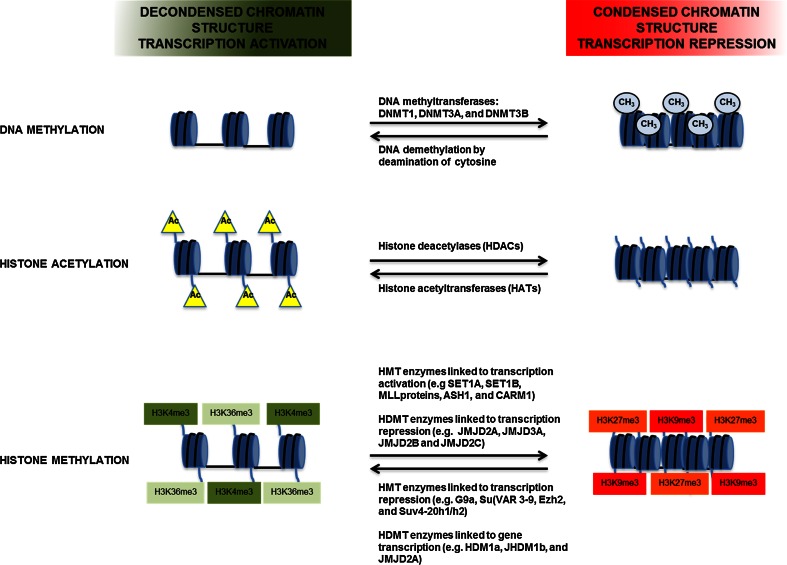
Schematic representation of the role of DNA methylation and histone acetylation and methylation in transcription regulation

Epigenetic mechanisms can be divided into four main categories on the basis of their mechanism of action: DNA methylation, covalent histone modifications, ATP-dependent chromatin remodeling (Fig. 1), and regulation by non-coding RNAs, including microRNAs (miRNAs). With the exception of miRNAs that act at the post-transcription level, all these mechanisms control gene expression through the modulation of chromatin structure and DNA-based biological processes, such as the binding of transcription factors to promoters and transcription elongation. Collectively, these modifications are called the epigenome.

Studies carried out over the last decade have demonstrated that some epigenetic players, such as histone deacetylases and miRNAs, have an important role in governing gene expression changes underlying heart failure [15, 23]. Moreover, recent reports support the involvement of epigenetic mechanisms, like DNA and histone methylation and ATP-dependent chromatin remodeling, in this pathology [17, 27, 46]. In this review, we illustrate the current knowledge regarding the role of epigenetics in heart failure.

## DNA methylation

DNA methylation was the first epigenetic mark to be discovered. It consists in the addition of a methyl group onto carbon 5 of the cytosine residues (5mC) located in CpG dinucleotides. CpG dinucleotides are distributed across the genome in “CpG islands”—genomic regions that have a high frequency of CpG dinucleotides—and regions with large repetitive sequences (e.g., satellite DNA, centromeric repeats, retrotransposon elements, and rDNA). In the human genome, CpG islands map preferentially to the 5′ end of genes and include the promoter; the majority of these (50–70 %) are methylated in somatic cells [36]. DNA methylation promotes gene silencing either by directly blocking the binding of transcription factors to DNA or by the binding of MBPs (methyl-binding proteins, e.g., MBD1, MBD2, MBD3, and MBD4), which can mediate gene repression through interaction with a co-repressor complex [18, 28].

Historically, DNA methylation was considered to be a stable epigenetic mark. However, high-resolution genome-wide mapping in pluripotent and differentiated cells has uncovered the dynamic nature of DNA methylation [26]. DNA methylation is catalyzed by DNA methyltransferase enzymes (DNMTs), such as Dnmt1, Dnmt3a, and Dnmt3b, which use S-adenosyl-methionine as the methyl donor. The mechanism of demethylation in mammals is unclear, but recent reports suggest that active demethylation can occur either by direct deamination on 5mC—mediated by activation-induced deaminase (AID)/APOBEC-family cytosine deaminases—or by oxidation of 5mC to 5-hydroxymethylcytosine (5hmC)—performed by ten–eleven translocation (TET) enzymes, which are Fe(II)/2-oxoglutarate-dependent oxygenases—followed by deamination catalyzed by base excision repair (BER) enzymes [6, 30, 33].

## Acetylation and methylation of histones

The fundamental unit of chromatin is the nucleosome, which is made up of a core of eight histones (two each of H2A, H2B, H3, and H4) around which a double-strand of DNA (147 nucleotides long) is wrapped in 1.75 superhelical turns. The amino-terminal tails of all eight core histones protrude through the DNA and undergo a variety of post-translational covalent modifications, such as acetylation, methylation, ubiquitylation, sumoylation, and phosphorylation, on specific residues. In the last few years, it has been demonstrated that acetylation and methylation are important epigenetic mechanisms involved in regulating key cellular processes, such as gene transcription, DNA replication, and DNA repair [5].

Histone acetylation occurs on Lys residues present in the tails of histone proteins H2B, H3, and H4, and signals for transcription activation, whereas hypoacetylated histone proteins are found in transcriptionally inactive regions. Acetylation is a dynamic process controlled by two families of enzymes: histone acetyltransferases (HATs) and histone deacetylases (HDACs), both of which include multiple enzyme classes whose expression and activity are finely regulated [40].

Unlike acetylation, histone methylation can be associated with either activation or repression of transcription, depending on the particular methylated Lys or Arg and on the degree of methylation (mono-, di-, and tri-methylation) [22]. Histone methylation is not a permanent histone modification, but rather a more dynamic process regulated by two classes of enzymes: histone methyltransferases (HMTs) and histone demethylases (HDMTs) [11]. An important aspect of epigenetic regulation is that combinations of DNA methylation and histone modifications create an ‘epigenetic code’ that defines genomic regions with different functions: transcriptionally active genomic regions are assembled into a chromatin structure that is poor in methylated DNA and rich in H3 histone that is acetylated and methylated on Lys 4 and Lys 36; in contrast, regions that are transcriptionally inactive are characterized by a high level of methylated DNA and H3 histone that is deacetylated and methylated on Lys 9 and 27 [38] (Fig. 1).

## ATP-dependent chromatin-remodeling complex

ATP-dependent chromatin-remodeling complexes (RCS) are specialized multi-protein complexes that use ATP to regulate gene expression by modulating the distribution of nucleosomes on DNA to make DNA-binding sites accessible to transcription factors, which can bind to DNA only when this is free from the nucleosome core. On the basis of its ATPase subunits, chromatin-remodeling complexes can be divided into four families of remodelers, referred to as SWI/SNF, ISWI, CHD, and INO80. In this section, we will focus on brahma-associated factor (BAF) complex, the vertebrate ortholog of SWI/SNF complex, which was identified initially in *Saccharomyces cerevisiae*. In mammals, there are 14 BAF subunits encoded by 25 genes; assembly of these subunits can form a hundred different BAF complexes, which have either brahma (Brm) or brahma-related gene 1 (Brg1) as the ATPase subunit. Certain BAF subunits are expressed only in specific cell types, defining the tissue- or cell-type-specific BAF complex [43].

BAF complex is involved in several cellular processes, including heart and muscle development [16]. The BAF180 subunit is required for normal heart chamber maturation and coronary development [42]. Deletion of Brg1 from endocardial cells in early stages of development causes defects in the morphology of the heart as a result of defective myocardial trabeculation [34].

## microRNAs

miRNAs are small RNAs (19–25 nucleotides long) that regulate a variety of biological processes, such as cell proliferation and differentiation, by silencing target mRNAs. Thus, in contrast to the above-described epigenetic mechanisms that modulate gene expression at the transcription level, miRNAs control gene expression at the post-transcriptional level. Each miRNA might regulate the expression of hundreds of target mRNAs. miRNA genes are organized within the genome in monocistronic or polycistronic units located in the introns of host (protein-coding or non-coding) genes or, rarely, in host exons, as well as in intergenic regions as independent genes [29].

**Fig. 2:**
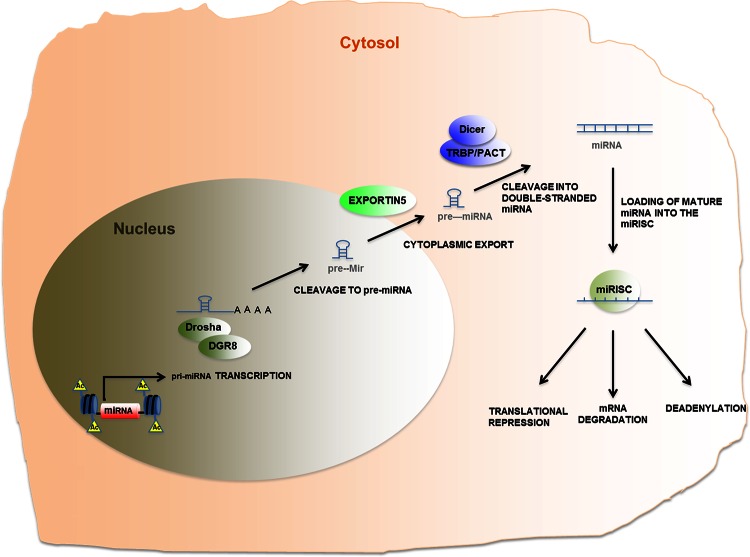
Schematic representation of micoRNA biogenesis

Many miRNAs are transcribed by RNA polymerase II to generate RNA precursors, called primary miRNAs, which are then processed into pre-miRNAs by the RNase III enzyme Drosha (Fig. 2). Pre-miRNA is exported to the cytoplasm by exportin-5, where another RNase III enzyme, Dicer, processes them into the mature miRNA. miRNAs interact with the protein Argonaute to form the RISC (RNA-induced silencing complex). RISCs bind to the 3′ UTR (untranslated region) of specific mRNA targets. At this point, either translation is blocked or the mRNAs are degraded [23].

## Interdependence of different epigenetic mechanisms

An important aspect of epigenetic regulation is cross-talk with other epigenetic mechanisms. Interesting examples of this come from recent studies on cancer cells, demonstrating that DNA methylation, histone modifications, and chromatin remodeling are linked to miRNA-mediated mechanisms: miR-203 is down-regulated by promoter CpG hypermethylation and this, in turn, enhances expression of the oncogenes ABL1 and BCR-ABL1 in various murine and human hematopoietic malignancies; miR-124a is repressed in acute lymphoblastic leukemia by hypermethylation of the promoter and histone modifications, including decreased levels of H3K4me3 and H3ac and increased levels of H3K9me2, H3K9me3, and H3K27me3, triggering the expression of the oncogene CDK6; and the expression Brg1, an important epigenetic regulator, is down-regulated by miR-199a-5p and -3p [1, 7]. Moreover, the miRNAs themselves are capable of controlling DNA methylation and histone modification by regulating the expression of genes that govern these epigenetic pathways. In skeletal muscle, miR-1 promotes myogenesis by targeting HDAC4, a transcriptional repressor of muscle gene expression [10]. The DNA methyltransferases DNMT3A and 3B are targets of miR-29, indicating that miRNAs can regulate also DNA methylation [13, 29].

These studies reveal that epigenetics is a complex network of mechanisms that work together in creating an “epigenetic landscape” for the regulation of gene expression at transcriptional and translational levels.

## Histone acetylation: an epigenetic mechanism involved in heart failure

**Fig. 3:**
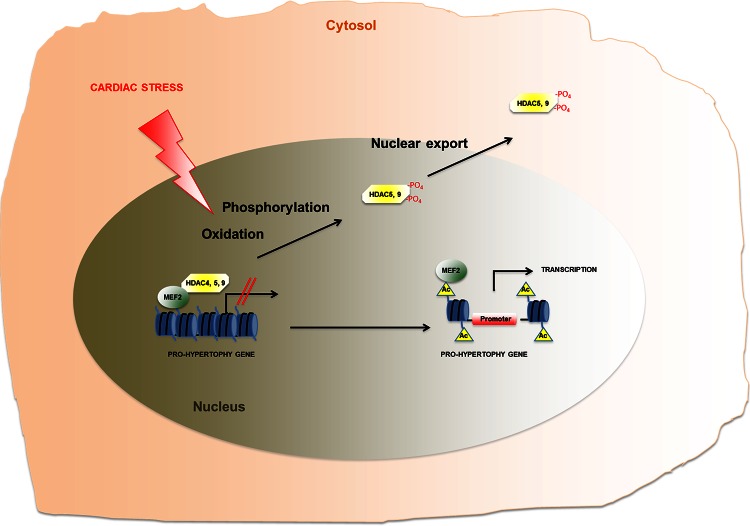
Schematic representation of the role of class II histone deacetylases (HDACs) in regulating gene expression re-programming in heart failure

Most research relating to the epigenetics of heart failure has focused on histone acetylation through the study of knockout mouse models of genes encoding HDACs. These studies have demonstrated that HDAC 5 and 9 (two class II HDACs) have anti-hypertrophic activity. Knockout mice of these genes show a major sensibility to the development of cardiac hypertrophy and a failure in responding to pro-hypertrophy stimuli, such as calcineurin activation and pressure overload. This is due to the ability of these enzymes to bind to and to inhibit Mef2c, a transcriptional factor that promotes gene expression of pro-hypertrophy genes. In response to a pro-hypertrophy stimulus, two stress-inducible kinases—calcium/calmodulin-dependent protein kinase (CaMK) and protein kinase D (PKD)—phosphorylate HDAC 5 and 9. Phosphorylated HDACs then bind 14-3-3, a chaperone protein that transports the HDACs from the nucleus to the cytoplasm. This causes the separation of HDAC 5 and 9 from Mef2c, which then is free to interact with p300, a histone acetyl transferase that promotes transcription [9, 15, 45, 47]. Another class II histone deacetylase involved in cardiac hypertrophy is HDAC4. In the heart under physiological conditions, HDAC4 functions as a repressor of MEF2 and serum response factor (SRF). Studies on molecular mechanisms involved in regulating the activity of HDAC4 in cardiac hypertrophy have revealed how oxidation of conserved cysteine and the proteolysis activity of protein kinase A (PKA) are two new regulatory mechanisms of this enzyme and, thus, of cardiac hypertrophy. Indeed, the activity of HDAC4 depends on its oxidation/reduction state: oxidation of HDAC4 causes a shuttling of the HDAC from the nucleus to the cytoplasm, de-repressing pro-hypertrophy genes; on the other hand, reduction of Cys-667/Cys-699 inhibits nuclear export of the HDAC independently of its phosphorylation state [2, 25] (Fig. 3). Moreover, PKA triggers proteolytic cleavage of HDAC4 to produce an N-terminal HDAC4 fragment (HDAC4-NT) that selectively inhibits the activity of MEF2 but not of SRF, antagonizing cardiac remodeling without affecting cardiomyocyte survival [4].

Recent studies also demonstrated that HDAC2 (a class I HDAC), unlike HDACs of class II, is involved in a pro-hypertrophy pathway. *Hdac2*-deficient mice are resistant to pro-hypertrophy stimulation; in contrast, mice overexpressing HDAC2 are over-sensitive to these stimuli. The pro-hypertrophy activity of HDAC2 is linked to its ability to repress the expression of *Inpp5f*, which encodes phosphatidylinositol-3,4,5-trisphosphate (PIP3) phosphatase, a negative regulator of the pro-hypertrophy PI3K–Akt–Gsk3β pathway [37].

As a whole, these studies suggest that different classes of HDAC are involved in different pathways that control hypertrophy: class II HDACs block the expression of pro-hypertrophy genes; on the contrary, HDAC2 which is a class I HDAC, is implicated in the block of expression of anti-hypertrophy genes.

The role of acetylation in heart failure is also supported by studies demonstrating that broad spectrum inhibitors of HDACs—such as trichostatin A (TSA) and sodium butyrate (NaB)—are able to prevent cardiac hypertrophy in vitro and how API-D, an inhibitor of class I HDACs, is capable of reducing cardiac hypertrophy in mice subjected to aortic constriction [3, 12, 21].

## DNA and histone methylation: new regulators of heart failure?

Although the role of histone acetylation has been largely investigated in heart failure, the involvement of other important epigenetic mechanisms, such as histone methylation and DNA methylation, remains poorly studied in this pathology.

Recent literature suggests an involvement of histone methylation in the regulation of cardiac hypertrophy at the transcriptional level. Keneda and colleagues [20], studying the genome-wide distribution of two types of histone methylation—tri-methylated histone H3H4 and tri-methylated histone H3K9—in rat and human heart, found that these epigenetic marks are altered in heart failure. A more recent report showed that JMJD2A/KMD4—a histone demethylase belonging to the JmjC domain-containing family JMJD2—is involved in cardiac hypertrophy: this enzyme catalyzes the demethylation of H3K9me3 and H3K36me3. Studies in mice have shown that a reduction of expression of JMJD2 in cardiomyocytes grants resistance to pressure overload induced by transverse aortic constriction (TAC), whereas *Jmjd2a*-transgenic mice display increased cardiac hypertrophy. It has been proposed that JMJD2A acts synergistically with SRF and myocardin in regulating FHL1, which is involved in mediating the hypertrophic response induced by TAC [46].

In addition, a study performed on PAX-interacting protein 1 (PTIP)—an essential co-factor for methylation of histone H3 at Lys 4—showed that a loss of H3K4me3 in adult cardiomyocytes is sufficient to cause the down-regulation of Kv channel-interacting protein 2 (Kcnip2), which is repressed in heart failure and has a role in arrhythmogenesis. Thus, H3K4me3 is required to maintain the transcription program in adult cardiomyocytes and could be involved in regulating gene expression changes in heart failure [35].

Finally, Bruneau and colleagues [39] demonstrated in a recent paper that an epigenetic signature defined by H3K27ac, H3K4me1, H3K4me3, and H3K27me3 is involved in governing gene expression changes underlying cardiomyocyte differentiation, suggesting a possible role for histone modification in the molecular etiology of congenital heart disease.

Regarding DNA methylation in heart failure, two reports recently gave support to the role of DNA methylation in cardiomyopathies. A genome-wide study of DNA methylation in end-stage cardiomyopathic patients’ hearts showed that the profile of this mark differed significantly from that of healthy hearts within the CpG islands of promoters and within gene bodies; moreover, methylation was significantly reduced at the promoters of up-regulated genes, but unchanged at the promoters of down-regulated ones [27]. In addition, Haas et al. [14] found an altered DNA methylation pattern in the myocardium of patients with idiopathic dilated cardiomyopathy, causing mis-expression of the genes for lymphocyte antigen 75 (*LY75*) and tyrosine kinase-type cell surface receptor HER3 (*ERBB3*), the zebrafish orthologs of which were found to be important for adaptive and maladaptive responses in heart failure.

These studies support a possible role for DNA and histone methylation in regulating gene expression changes underlying heart failure. However, they do not clearly demonstrate whether these epigenetic marks are involved in regulating gene expression in cardiac hypertrophy or at which stage of the pathology they are involved in.

## The chromatin-remodeling factor Brg1 is a key player in heart failure

A role for chromatin remodeling in regulating gene expression changes underlying heart failure is supported by recent studies on BRG1, an ATPase subunit of BAF. Mice lacking this protein are more resistant than wild type mice to a pro-hypertrophy stimulus, such as TAC. Moreover, the expression of this protein was found up-regulated in the heart of certain hypertrophic cardiomyopathy patients, with a correlation between expression level and disease severity. Brg1 plays a key role in the switch from a fetal myosin heavy chain (i.e., β-MHC or Myh7) to the adult MHC (α-MHC or Myh6) isoform during cardiac hypertrophy. Brg1 is expressed in embryonic heart but not in adult cardiomyocytes. However, pro-hypertrophy stimuli induce re-expression of this protein, which then interacts with its embryonic partners HDAC and PARP, forming two molecular complexes responsible for the pathological shift from adult to fetal MHC isoforms: the Brg1–PARP–HDAC complex binds to the α-MHC promoter to repress α-MHC transcription, whereas the Brg1–PARP complex activates the transcription of β-MHC by binding to its promoter [17].

## miRNAs are key regulators of heart failure

With respect to covalent modification of DNA and histones, the role of miRNAs in heart failure has been more extensively investigated. Over the last few years, a handful of reports have demonstrated an important role in the pathogenesis of heart failure. The expression of many miRNAs has been found altered in animal models of heart failure and in human cardiac patients [23]. Interestingly, the miRNA signature of failing heart has a similarity to that of fetal cardiac tissue; this is in accordance with the idea that during heart failure, the fetal-gene expression program is reactivated. It has also been suggested that for each heart pathology there is a specific miRNA signature. Indeed, a genome-wide study on miRNA expression demonstrated how three human heart pathologies—aortic stenosis, dilated cardiomyopathy, and ischemic cardiomyopathy—have 43 out of 87 tested miRNAs differentially expressed in at least one of these disease groups [19].

Functional studies performed in vitro and in vivo have demonstrated the key role of miRNAs in driving gene expression change during heart failure. In fact, overexpression of miR-23a, miR-23b, miR-24, miR-195, or miR-214 (all found up-regulated with disease) individually induced cardiac hypertrophy in neonatal cardiomyocytes; transgenic miR-195 mice were found to develop dilated cardiomyopathy; and overexpression of miR-133 (found down-regulated with disease) inhibited cardiac hypertrophy, whereas miR-133 deletion promoted cardiac hypertrophy in vivo and in vitro [8, 23]. Altogether, these studies suggest that miRNAs represent a good therapeutic target and/or have a diagnostic significance for heart failure.

## Conclusion

The above-described studies lend support to an important role of epigenetics in the etiology of heart failure and, in particular, cardiac hypertrophy. However, many questions remain open, such as: (1) which genomic regions are regulated by epigenetic mechanisms; (2) what is the biological significance of the combination of DNA methylation and histone modifications (the epigenetic code); (3) how do the epigenetic mechanisms related to DNA methylation, histone modifications, chromatin remodeling, and miRNAs interact; (4) how are the other cardiac cell types (e.g., cardiac fibroblasts and endothelial cells), in addition to cardiomyocytes, affected by epigenetic changes in heart failure; and (5) how does the epigenome modulate the effect of the environment (e.g., diet, smoking, stress) in the etiology of heart failure.

In recent years, the study of epigenetics in several fields, such as cancer and cell differentiation, has received great benefit from the development of next-generation high-throughput DNA sequencing (NGS) technologies, which enable the sequencing of hundreds of millions of short DNA fragments in a single run. The combination of these DNA sequencing (seq) methods with traditional ways of studying epigenetic mechanisms, such as chromatin immunoprecipitation (ChIP) and DNA bisulfite conversion, has led to the development of ChIP-seq, methyl-ChIP-seq, and bisulfite sequencing of DNA, techniques that generate high-resolution genome-wide profiles for DNA methylation and histone modifications [31, 32]. Moreover, NGS technologies applied to RNA (RNA-seq) have recently provided a more powerful means for the analysis of transcript expression, including that of miRNAs, and for the identification of new non-coding RNAs that could play a role in epigenetics [41]. The implementation of these methodologies in the cardiovascular field will certainly help to answer the above questions and, thus, to shed light on the epigenetic events underlying transcription changes that accompany heart failure.
